# Astrocytic atrophy as a pathological feature of Parkinson’s disease with LRRK2 mutation

**DOI:** 10.1038/s41531-021-00175-w

**Published:** 2021-03-30

**Authors:** Paula Ramos-Gonzalez, Susana Mato, Juan Carlos Chara, Alexei Verkhratsky, Carlos Matute, Fabio Cavaliere

**Affiliations:** 1 Department of Neurosciences, University of the Basque Country UPV/EHU, Leioa, Spain; 2 Achucarro Basque Center for Neuroscience, Leioa, Spain; 3 Centro de Investigación Biomédica en Red sobre Enfermedades Neurodegenerativas (CIBERNED), Madrid, Spain; 4 Biocruces, Bizkaia, Barakaldo, Spain; 5 Faculty of Biology, Medicine and Health, The University of Manchester, Manchester, M13 9PT UK; 6 Sechenov First Moscow State Medical University, Moscow, Russia

**Keywords:** Brain, Parkinson's disease

## Abstract

The principal hallmark of Parkinson’s disease (PD) is the selective neurodegeneration of dopaminergic neurones. Mounting evidence suggests that astrocytes may contribute to dopaminergic neurodegeneration through decreased homoeostatic support and deficient neuroprotection. In this study, we generated induced pluripotent stem cells (iPSC)-derived astrocytes from PD patients with LRRK2^(G2019S)^ mutation and healthy donors of the similar age. In cell lines derived from PD patients, astrocytes were characterised by a significant decrease in S100B and GFAP-positive astrocytic profiles associated with marked decrease in astrocyte complexity. In addition, PD-derived astrocytes demonstrated aberrant mitochondrial morphology, decreased mitochondrial activity and ATP production along with an increase of glycolysis and increased production of reactive oxygen species. Taken together, our data indicate that astrocytic asthenia observed in patient-derived cultures with LRRK2^(G2019S)^ mutation may contribute to neuronal death through decreased homoeostatic support, elevated oxidative stress and failed neuroprotection.

## Introduction

Parkinson’s disease is the second most common neurodegenerative disorder with unknown aetiology^1^. Age is the principal risk factor for PD, which affects around 1% of people older than 65 years^2^. The progressive death of dopaminergic neurones in the substantia nigra *pars compacta* (SNpc) and the appearance of protein deposits in a form of Lewy bodies (LB) mainly composed by α-synuclein (α-syn) represent two major histopathological hallmarks of PD^3–5^. Although the disease is mostly idiopathic, 10% of the cases appear related to specific mutations in different genes. The G2019S mutation in Leucine Rich Repeat Kinase 2 (*LRRK2*) gene is the most common cause of the familial PD^6^. This mutation leads to an idiopathic phenotype of the disease albeit, in certain cases, with the absence of LB^7^. The G2019S mutation is the most frequent pathogenetic mutation in the overall LRRK2-PD population^8^. This mutation occurs in the kinase domain of LRRK2, leading to an increase in the activity of the enzyme^9^, which has been shown to affect mitochondrial functionality, cytoskeletal dynamics, response to reactive oxygen species (ROS) production, and autophagy^10,11^.

Fibroblasts from PD patients carrying the G2019S mutation showed abnormal mitochondrial morphology^12^. Similarly, overexpression of wild-type LRRK2 in SH-SY5Y neuroblastoma cells caused mitochondrial fragmentation, which was further enhanced when the R1441C and G2019S mutations were expressed^13^. Overexpression of LRRK2^G2019S^ mutation in SH-SY5Y cells causes mitochondrial uncoupling, leading to membrane depolarisation and increased oxygen consumption^14^. The LRRK2^G2019S^ mutation also delays the digestion of dysfunctional mitochondria and the initiation of mitophagy^15^.

Numerous studies have established a connection between LRRK2 and both microtubules (MTs) and filamentous actin (F-actin). A high-throughput screening performed to reveal LRRK2 interactome identified proteins involved in actin filament assembly, organisation, rearrangement, and maintenance, suggesting that the biological function of LRRK2 is linked to cytoskeletal dynamics^16^. The same study demonstrated that LRRK2 binds to F-actin and modulates F-actin assembly in mouse primary dopaminergic neurones in vitro. This suggests that morphological changes and abnormalities in neurites outgrowth and branching may be consequences of LRRK2-modulation of cytoskeletal dynamics.

Thus, analysis of PD pathogenesis has been mostly focused on the mechanisms underlying ventral midbrain dopaminergic neurones (vmDAn) degeneration and death. Neuronal survival, however, is defined by multiple neuroprotective mechanisms expressed in astrocytes, the principal homoeostatic and defensive cells of the central nervous system^17–19^. Astrocytes density in the SN is relatively low^20^, which may strain their ability to adequately support and protect neurones. In PD, in contrast to other α-synucleopathies, astrocytes do not mount reactive astrogliosis^21^, an evolutionary conserved defensive response; rather, astrocytes become dysfunctional and lose their protective capabilities^22^. Astroglial atrophy, asthenia and loss of homoeostatic and protective function contribute to several neurodegenerative and psychiatric diseases^23^. A recent study demonstrated that the treatment of LRRK2^G2019S^ transgenic mice with α-syn increases the expression of endoplasmic reticulum (ER) stress proteins in astrocytes thus affecting neurites length and neuronal viability, supporting the idea that ER stress in PD astrocytes can aggravate neuronal damage^24^.

In this study, we have generated and characterised human iPS-derived astrocytes (hiA) from PD patients carrying LRRK2^G2019S^ mutation. These PD astrocytes display an atrophic morphology with decreased complexity, as well as altered mitochondrial functionality that results in higher basal protein oxidation. As a consequence, PD astrocytes show reduced mitochondrial metabolism and increased glycolytic activity. Overall, we suggest that LRRK2^G2019S^ mutation in astrocytes induces mitochondrial unbalance, leading to cell autonomous and non-autonomous damage that ultimately translates to or exacerbates neurodegeneration. Our results highlight an improvement of astroglial functionality as a relevant therapeutic target.

## Results

### Generation of patient-derived astrocytes from dermal fibroblasts

**Fig. 1:**
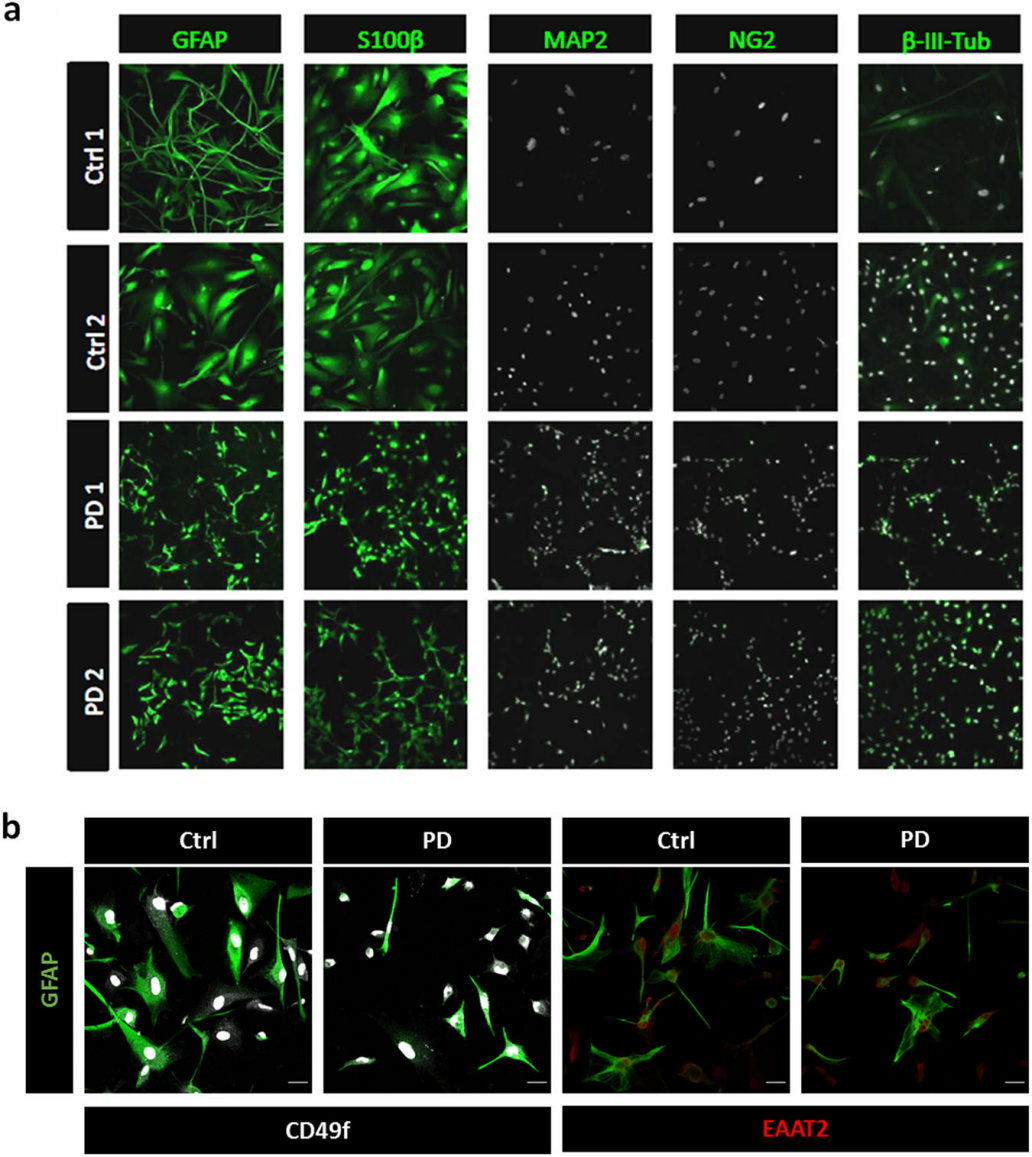
Astrocytic marker expression in hiA. **a** hiA were maturated for 60 days in LN211/LN111 and fixed for immunofluorescence. All cell cultures, from healthy (Ctrl1-2) and patient (PD1-2) donors, were positive for GFAP and S100β expression, whereas neuronal (MAP2 and β-III-Tub) and non-astrocyte-glial (NG2) markers were nearly absent. White staining shows nuclei labelling by DAPI. **b** hiA co-immunostaining of GFAP with CD49f and EAAT2 in healthy (Ctrl) and patient (PD) donors. The picture is representative of two Ctrls and two PD cell lines. Scale bar is 25 μm. Photographs are representative of at least five experiments.

Skin fibroblasts from two patients with LRRK2^G2019S^ mutation and two healthy donors (Supplementary Table 1) were reprogrammed and differentiated to mature astrocytes. Fibroblast were reprogrammed using the episomal Sendai viral vector bearing the Yamanaka factors Klf4-Oct3/4-Sox2 (KOS), L-Myc and Klf4 (see Supplementary Fig. 1 for protocol details). Fibroblasts were expanded in Geltrex until they formed colonies positive for the pluripotent markers Sox2, Oct4 and Nanog (Supplementary Fig. 2). To further potentiate the formation of iPSC, colonies were picked and expanded for 2–4 days in human recombinant laminin-521 (LN521). LN521 is normally expressed in the human embryo at the inner cell mass and replicates the human stem cell niche in vitro stabilising pluripotent gene expression. At this stage and before neural induction, iPSC can differentiate to the three germ layer as evidenced by the expression of Neurone-specific class III β-tubulin (Tuj1 for ectoderm), smooth muscle actin (SMA for mesoderm) and alpha-Fetoprotein (AFP for endoderm) (Supplementary Fig. 2). To induce the differentiation to NSC, neural rosettes were cultured with a 50%:50% mixture of laminin 211 (LN211) and laminin 111 (LN111) coating. Unlike LN521, these two laminins are mostly expressed in extra-embryonic membranes and promote cell differentiation. In our culture conditions, NSCs were differentiated to astrocyte progenitor cells in 21 days. Subsequently, astroglial precursors were further differentiated into mature astrocytes (see Material and Methods) while maintaining the coating with LN211/LN111 (50%:50%). After 60–75 days of maturation, cells were fixed and stained with the astrocyte marker GFAP. Maturation efficiency was evaluated by cytofluorimetry assay (Supplementary Fig. 3) demonstrating 95%–98% of astrocyte differentiation. Astrocyte differentiation was also confirmed by immunofluorescence with antibodies to GFAP and S100B, whereas expression of MAP2 and β-III tubulin (for neurones) and NG2 (for non-astrocyte glia) was absent or minimal (Fig. 1a). The hiA also expressed the functional markers EAAT2 (glutamate transporter) and CD49f, with undetectable differences between healthy subjects and PD donors (Fig. 1b). All generated lines from the four donors (healthy and PD) displayed neither genetic nor structural variations in somatic and sex chromosomes as demonstrated in Supplementary Fig. 4.

### Morphology of PD astroglia

**Fig. 2:**
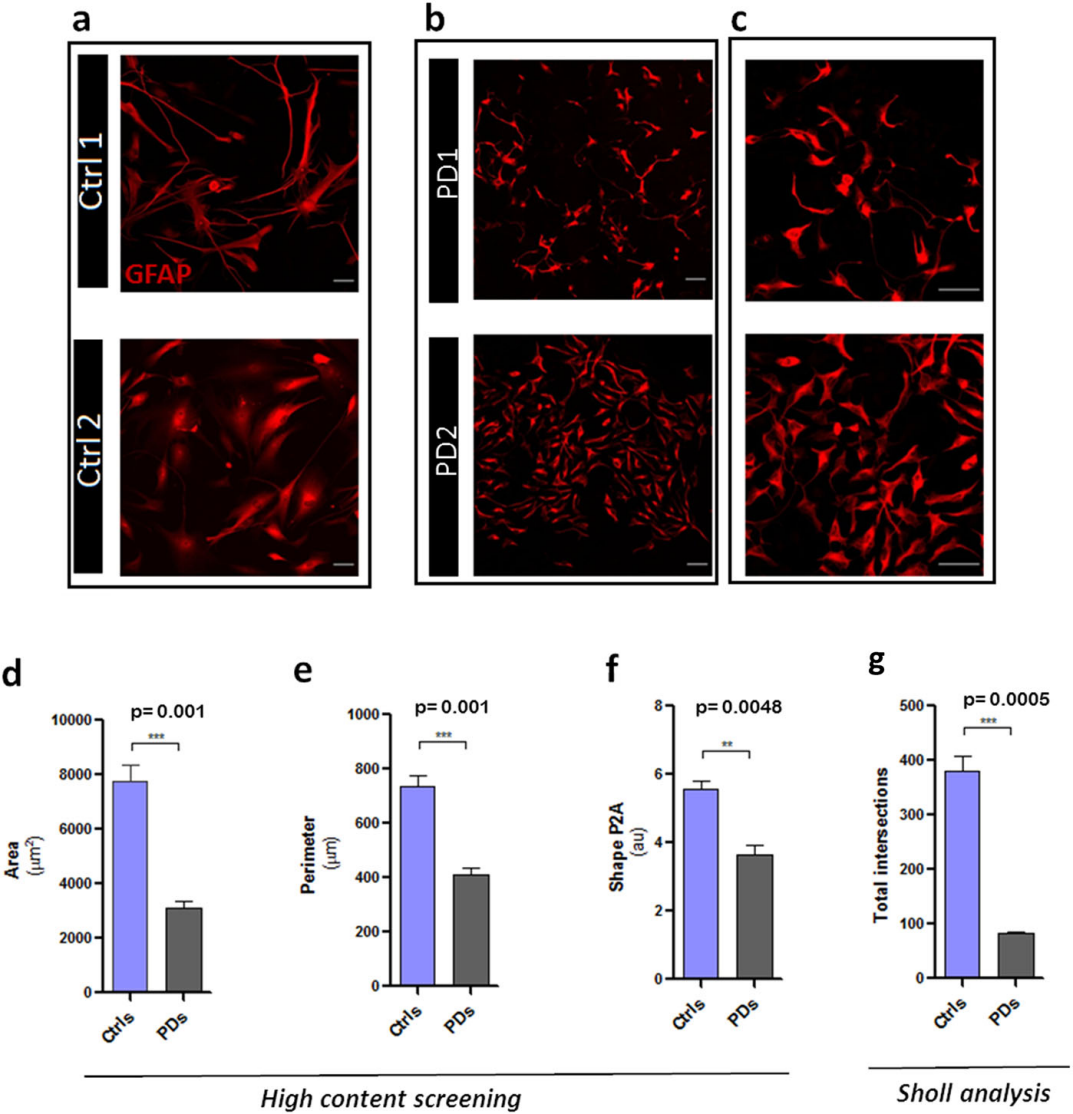
Cell morphology analysis of PD astroglia. Cells from healthy (Ctrl1-2 in **a**) and patients (PD1-2 in **b**, **c**) donors were fixed after 60 days of maturation and stained with GFAP antibodies. Images in **c** illustrate a higher magnification of a subfield in **b**. Morphological analysis was performed by *high-content screening* (**d**–**f**) and Sholl analysis (**g**) as described in Materials and Methods. **d**–**f** histograms showing astrocyte area (as a means of squared μm in **d**), perimeter (as a means of linear μm in **e**) and complexity (as a means of arbitrary units-au- of shape P2A in **f**). In **g** is expressed the total number of intersections with concentric rings of Sholl grid (5 μm apart) after GFAP immunostaining of controls and PD astrocytes. Data are presented as mean values ± SEM with *n* = 4.

We observed a striking difference in the morphology between healthy and PD astrocytes (Fig. 2a–c). The surface area and perimeter of GFAP-positive profiles of PD-derived astrocytes were, respectively, 60% and 45% smaller when compared to healthy cells, as measured by high-content screening (Fig. 2d–e). Similar data (decrease in surface area and perimeter by 69% and 50%; data not shown) were obtained by manual measurements using the image software Fiji. Astrocytes from PD patients showed a lower complexity with significant reduction in number or complete absence of primary and secondary processes (Fig. 2f), as evidenced by high-content screening analysis (35% lower than healthy astrocytes), suggesting a decreased structural capacity for supporting neurones. Decreased complexity of PD-derived astrocytes was also confirmed by Sholl analysis. As shown in Fig. 2g, astrocytes derived from PD donors exhibit 61% less intersections. Morphological atrophy in PD cultures was not detected in fibroblasts; moreover, we did not detect differences during the iPSC colony formation, but only after astrocyte differentiation (data not shown), suggesting a specificity for the astrocytic phenotype.

### Functional characterisation of PD astrocytes

**Fig. 3:**
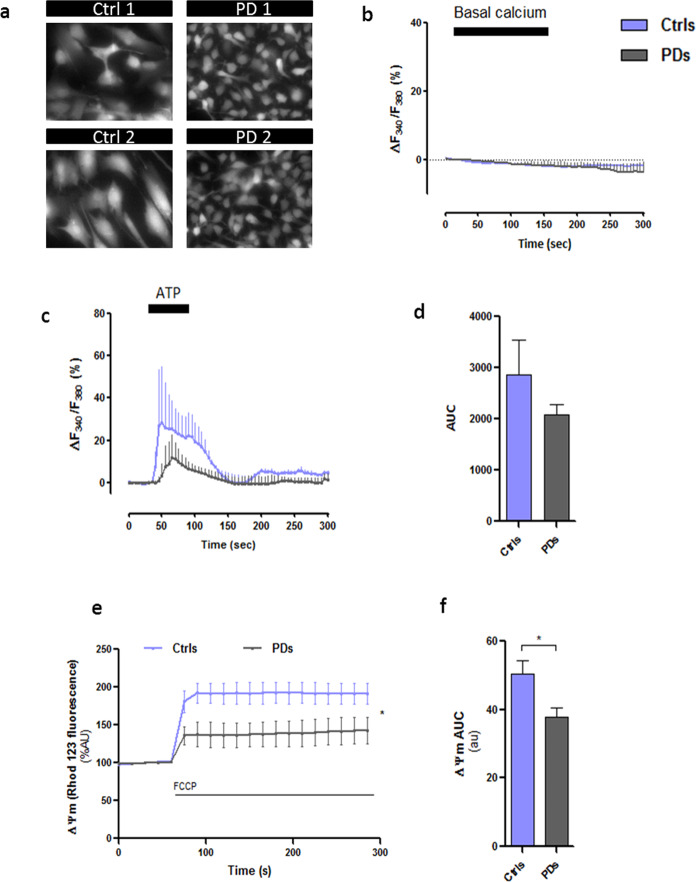
Cytosolic Ca^2+^ responses to ATP and FCCP in human astrocytes. **a**–**b** Time-courses show basal cytosolic Ca^2+^ responses in control and PD hiA loaded with fura-2 (*n* = x-y cells). **c** Ca^2+^ responses evoked by ATP (100 μM) in hiA (*n* = x-y cells). **d** Comparison of the area under the curve (AUC) calculated for each experimental condition (*n* = 3 cultures). **e**–**f** Measurement of mitochondrial membrane potential in hiA. *p = 0.0365. One-way ANOVA followed by Newman–Keuls tests. Ctrls, control. PDs Parkinson´s disease. Data are presented as mean values ± SEM with *n* = 3.

We next characterised functional properties of hiA. Astroglial function and reactivity are tightly integrated with the dynamics of cytosolic and mitochondrial Ca^2+^ concentrations controlling bioenergetics; abnormal astrocytic Ca^2+^ signalling is increasingly recognised as a key process in neurodegenerative conditions^25–27^. Thus, we analysed Ca^2+^ dynamics in PD astroglia. Neither healthy nor PD astrocytes generated spontaneous Ca^2+^ transients under our experimental conditions and we found no difference in resting cytoplasmic Ca^2+^ concentration ([Ca^2+^]_i_) between healthy and PD hiA (Fig. 3a–b). Application of 100 μM ATP (an archetypal activator of astroglial Ca^2+^ signalling) evoked transient [Ca^2+^]_i_ elevation (Fig. 3c–d) confirming the presence of functional purinergic receptors coupled to astrocytic Ca^2+^ signalling machinery. In PD astrocytes we observed a tendency (which did not reach the level of significance) of reduction in amplitude and integral of Ca^2+^ transients in response to ATP (Fig. 3c–d). It has to be noted however, that control hiA lines tested in this study displayed marked differences in the amplitude of ATP-induced Ca^2+^ transients. We further tested mitochondrial membrane polarisation by imaging the quenching of the mitochondrial membrane potential probe Rhodamine 123 in the presence of FCCP, which revealed significant differences between control and PD hiA lines (*p* = 0.0365); (Fig. 3e–f). Taken together, these finding confirm that hiA, with differences between healthy and PD astrocytes, express functional receptors for ATP, typical astrocytic Ca^2+^ signalling machinery; the PD-derived astrocytes also demonstrated signs of mitochondrial malfunction.

### Mitochondrial impairment in PD astrocytes

**Fig. 4:**
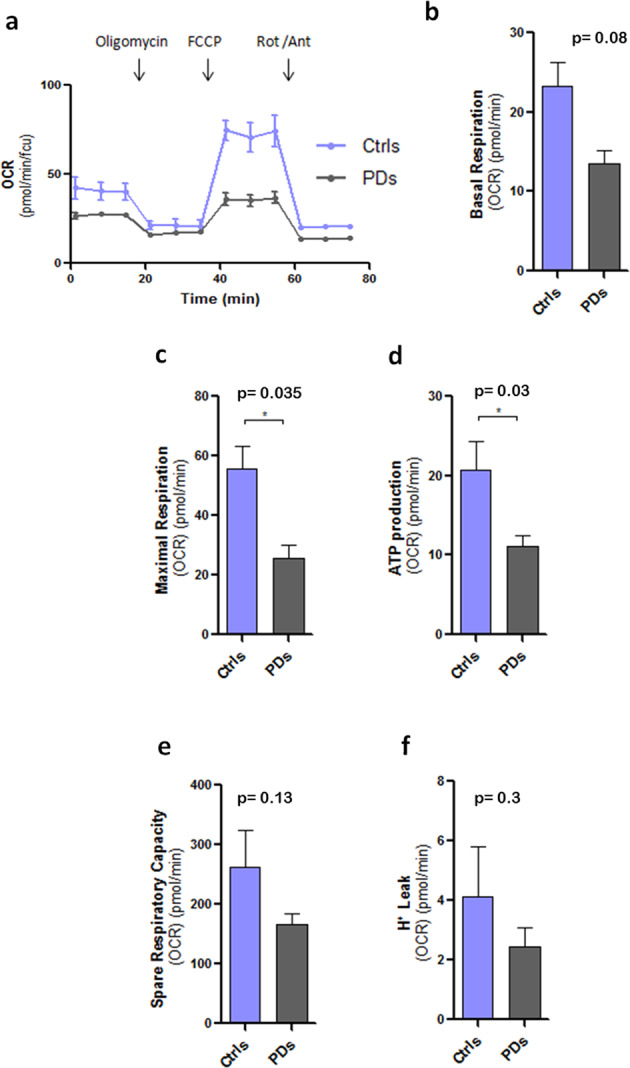
Mitochondrial metabolism and respiration. **a** Oxygen consumption rates (OCRs) of Ctrl and PD astrocytes. Oligomycin, FCCP and rotenone (Rot) were added, respectively, after 20, 40, and 60 min as respiratory chain blockers. OCRs are expressed in **b**–**f** as pmol por minute after cell viability normalisation with calcein staining. **b** Basal respiration, **c** maximal respiration and **d** ATP production are reduced in PD astrocytes compared to the controls. **e** Spare respiratory capacity and **f** H^+^ Leak do not show statistically significant changes (*n* = 4). Statistical analysis was performed using one-way ANOVA. Data are presented as mean values ± SEM.

**Fig. 5:**
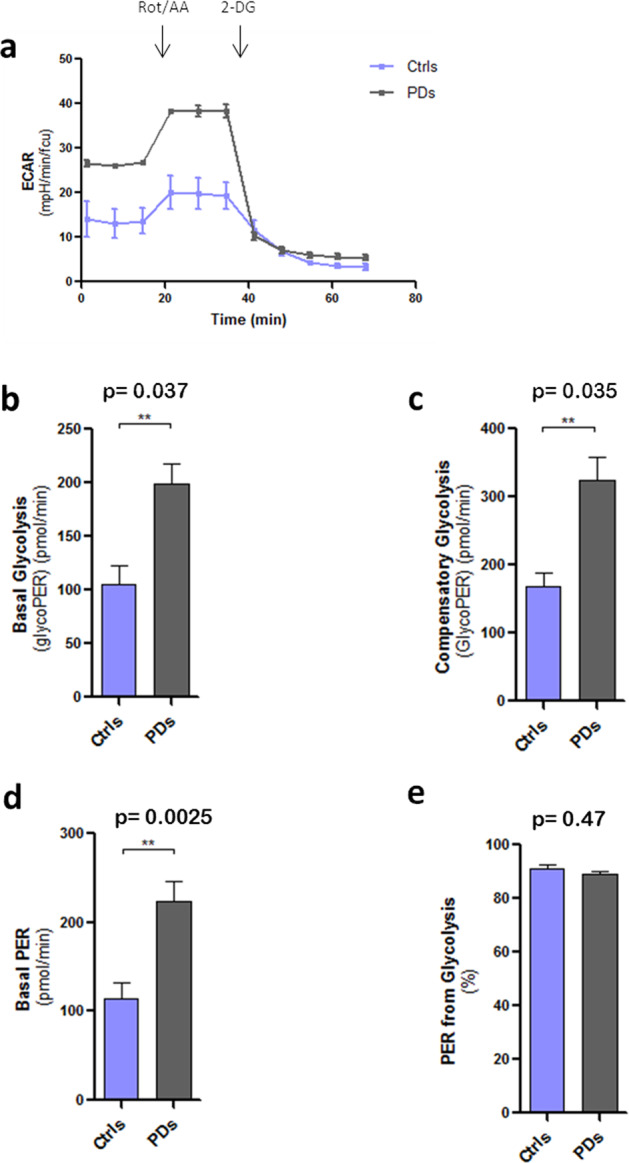
Glycolytic activity. **a** Extracellular acidification rate (ECAR) of Ctrl and PD astrocytes. **b** Basal glycolysis, expressed as pmol/min of glycolytic proton efflux rate (PER), **c** Compensatory glycolysis and **d** Basal PER, expressed as pmol/min of PER, are increased in PD astrocytes compared to the controls. **e** PER from glycolysis is used as an internal control and it is similar in the four lines (*n* = 3). Statistical analysis was performed using one-way ANOVA. Data are presented as mean values ± SEM.

It is well known that LRRK2 protein interacts with mitochondrial membranes and affects mitochondrial respiration^13,28^. We, thus, analysed mitochondrial metabolism in healthy and in PD astrocytes using Seahorse technology. We first measured the mitochondrial oxygen consumption rate (OCRs) of hiA in a live-cell metabolic assay (Fig. 4a). PD astrocytes showed lower OCRs in both basal (Fig. 4b) and maximal (Fig. 4c) respiration paradigms, when compared to healthy cells. PD astrocytes also produced less ATP (Fig. 4d). We did not, however, observe significant differences between healthy and PDs astrocytes in terms of spare respiratory capacity or proton leak (Fig. 4e–f). Consistent with a mitochondrial respiration deficit, PD astrocytes displayed increased glycolytic capacity as determined by changes in the extracellular acidification rate (ECAR) (Fig. 5a), both in basal (Fig. 5b) and in compensatory glycolysis (Fig. 5c). Similarly, basal proton efflux rate (PER, the measure of extracellular acidification, Fig. 5d), but not the PER derived from glycolysis (Fig. 5e), was increased in PD astrocytes. Collectively these results indicate that astrocytes in PD switch from oxidative phosphorylation to the aerobic glycolytic respiration.

**Fig. 6:**
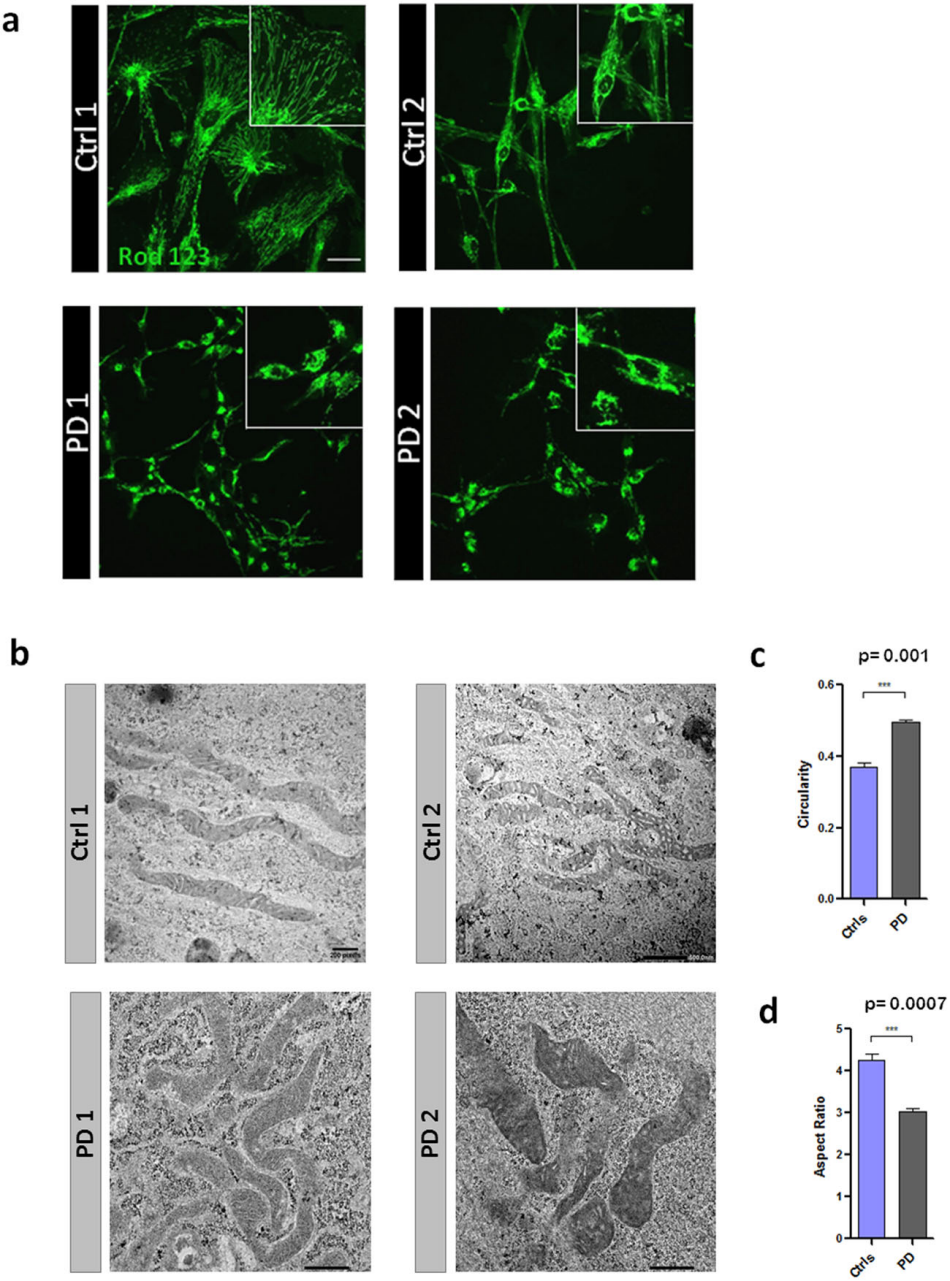
Analysis of mitochondrial morphology. **a** Mitochondrial staining with Rhodamin 123 of hiA cultures from healthy (Ctrl1-2) and patients (PD1-2) donors . Images were taken with the confocal microscope Leica TCS STED CW SP8. Squared inlets represent a higher magnification of the field. Scale bar 20 μm. **b** Representative images of mitochondrial ultrastructure in Ctrl and PD astrocytes. **c** Circularity is measured considering 1 as the perfect circle and **d** aspect ratio (ratio of circularity vs. elongation) reveal a more rounded shape in PD astrocyte mitochondria compared to the control. More than 100 mitochondria were analysed for each line. Statistical analysis was performed using one-way ANOVA. Data are presented as mean values ± SEM.

Mitochondrial malfunction is frequently associated with aberrant morphology^29^ and, therefore, we compared intracellular distribution and the ultrastructure of mitochondria in healthy and PD astrocytes. Mitochondrial distribution and gross morphology was visualised by staining with Rhodamin 123. In healthy astrocytes, mitochondria were elongated and interconnected, forming a homogenous network distributed throughout the entire cytoplasm, being present in the soma and in the principal processes (Fig. 6a). In contrast, PD astrocytes had fewer mitochondria, which were apparently more fragmented and mainly concentrated in the perinuclear region; in addition mitochondria were absent from short processes (Fig. 6a). The very same distribution pattern was observed after staining with Mitotracker (Supplementary Fig. 5), which demonstrates evident perinuclear concentration of mitochondria in PD astrocytes. Ultrastructural analysis of mitochondria (Fig. 6b), revealed further differences between the healthy and PD astrocytes. The measurement of the circularity, usually used as an index of ROS production, demonstrated that mitochondria in PD astrocytes were more rounded than in the control cells (Fig. 6c). Accordingly, the Aspect Ratio (the major axis divided the minor axis of the mitochondria) was higher in healthy astrocytes indicating the presence of more elongated mitochondria.

**Fig. 7:**
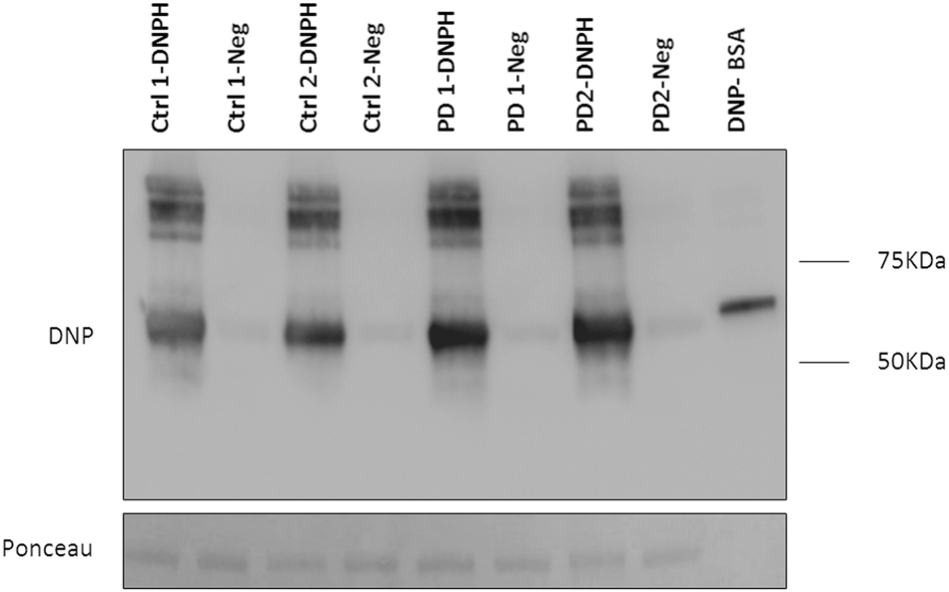
Detection of oxidised proteins in total astrocyte protein. Total proteins from hiA cultures were extracted after 60 days of in vitro maturation. Oxidised proteins are visualised after Western blot analysis as the conversion of the 2,4-dinitrophenol (DNP) to 2,4-dinitrophenylhydrazine (DNPH). Each sample is loaded as a negative control (Neg) with non-derivatised procedure. DNPH levels were normalised with total proteins stained with Red Ponceau.

According to the hypothesis by which fragmented mitochondria are associated with higher levels of ROS^29,30^, we investigated astrocytic metabolic profile. Using Oxyblot analysis, we measured the carbonyl groups of total proteins extracted from healthy and diseased lines as a readout of the oxidative status of the proteins. We found higher amount of oxidised proteins (32%) in PD astrocytes when compared to the controls (Fig. 7), suggesting a basal oxidative status of astrocytes in PD higher than in healthy astrocytes. We may conclude, therefore, that LRRK2^G2019S^ mutation corresponds to a general mitochondrial dysfunction in astrocytes, with impaired mitochondrial respiration, cellular localisation and mitochondrial ultrastructure.

## Discussion

To study human diseases, the “humanised” experimental preparations are essential; even the most sophisticated animal models of human pathologies are not faithful replicas^31,32^. In this paper, we analysed morphological characteristics and metabolic profile of astrocytes derived from iPSCs generated from PD patients bearing the LRRK2^G2019S^ mutation. Using different combinations of several laminins coating, we obtained almost homogeneous cultures of human astrocytes (95%–98%). The purity of cultures was confirmed by citofluorimetry analysis (Supplementary Fig. 3). We simulated the physiological conditions occurring during the embryonic development by mixing laminins LN521 and LN511. Both these laminins are expressed in the inner cell mass and support survival and self-renewal of the pluripotent stem cells through the interaction with α6β1 integrin and PI3/Akt activation^33,34^. In contrast, mature astrocytes express LN111 and LN211^35,36^; activation of these two laminins supports cell differentiation and specialisation, such as, for example, the maintenance of the blood-brain barrier integrity^36^.

The role of astrocytes in pathological progress of PD is yet to be fully characterised. Recent works conducted on some inflammatory experimental paradigms have suggested two subtypes of astrocytes, A1 and A2 with neurotoxic and neuroprotective profiles^37^. The A1/A2 dichotomy has been based on correlative analysis of limited number of genes detected for specific conditions in the in vitro settings. This binary polarisation has not been confirmed^38–43^ and, similarly to once popular, but now discarded, M1/M2 microglial polarisation concept, has been repudiated by neuroglial community^44^.

Nonetheless, early in vitro experiments have clearly demonstrated that astrocytes protect and support survival of dopaminergic neurones^45^. Subsequent studies revealed that functional exhaustion and loss of astroglial homoeostatic support are dominant glial contribution to the PD, and the special definition of “dysfunctional” astrocytes has been introduced^22,46^. Furthermore, analysis of post-mortem samples of susbtantia nigra obtained from PD patients demonstrated significant decrease in expression of astroglial markers compared to healthy controls^47,48^; these findings being in general agreement with our concept of astroglial atrophy linked to the disease. Astroglial asthenia, atrophy and loss of homoeostatic and neuroprotective capacities were noted in aging^40^ and in various neurodegenerative and neuropsychiatric diseases^23,49^; astroglial atrophy thus represents a defined class of astrogliopathies^50^.

Obtaining an almost pure population of iPSC-derived astrocytes allowed us to study human astrocytes bearing pathophysiological signature. We have found a prominent aberrant morphology of astrocytes derived from PD LRRK2^G2019S^ patients. Differentiated astrocytic cultures, obtained from both healthy and PD subjects, expressed classical astrocyte markers (GFAP, S100B, CD49f and EAAT2). The PD astrocytes, however, were characterised by substantially smaller area and perimeter; they also show diminished complexity of primary and secondary processes as evidenced by high-content screening and Sholl analysis. These morphological changes do not represent culture artefact because this atrophy was observed only in fully differentiated astrocytes and not at preceding derivation stages. Previously published study of iPSCs-derived astrocytes with LRRK2^G2019S^ mutation^51^ did not found conspicuous morphological changes, although astrocytic appearance was not analysed in detail. We assume that the use of specific feeder layers (laminins) and smaller number of cell passages in our protocol (differentiation to astrocyte proceeds with weekly passages) diminishes cell reactivity thus reliably revealing cell morphology. Our findings of pronounced morphological atrophy in human iPSCs parallel recent demonstration of similar morphological atrophy in iPSC-derived astrocytes generated from early familial and late sporadic AD patients^52^. Morphological atrophy of astrocytes is arguably associated with neuronal damage (due to failed homoeostatic support) and aberrant synaptic connectivity manifest in neurodegenerative and psychiatric diseases (for a review see ref. ^50^). In many cases, astrocytic atrophy precedes cell death and neuronal degeneration. For example, in acute excitotoxic neurodegeneration and ALS, morphological aberrations are accompanied with the down-regulation of glutamate transporters and increased excitotoxicity^18^. Morphological atrophy in AD has been described in animal models^54–56^, in human iPSC-derived astrocytes from patients^53^, in deprenil-based brain imaging in patients^57^, and in post-mortem brain at late stages of the disease (Rodriguez and Verkhratsky, unpublished results). In our culture conditions atrophic astrocytes from PD patients showed normal viability, as demonstrated by expression of classical markers (Fig. 1) and by physiological [Ca^2+^]_i_ dynamics (Fig. 3). At the same time PD astrocytes demonstrate reduced mitochondrial functionality (Figs 4–6). Mitochondrial aberrations and morphological atrophy may explain why astrocytes in PD with LRRK2^G2019S^ mutation fail to support and protect neurones. This loss of function became even more evident in specific brain regions, specifically for *substantia nigra pars compacta* and striatum, where astrocytic density is lower compared to other regions^20^. Furthermore, astrocytes from *substantia nigra* seem to be unusually vulnerable to ischaemic attack^58^ and oxidative stress^59^. Loss of astroglial support may act as an exacerbating factor in neurodegenerative process; chronically malfunctional astroglia was suggested to contribute to death of dopaminergic neurones^60^. It is early to conclude that astrocytic atrophy is the hallmark of PD until the same astrocytic atrophy is characterised in situ in patient’s brain tissues or in astrocytes derived from other mutations. Detailed analysis of astrocyte morphology should be performed in other regions of the brain that are related with dopaminergic degeneration (e.g., subthalamic nucleus or globus pallidum) and not only in degenerating regions where astrocytes are pathologically remodelled.

Mutation in the LRRK2 gene may be specifically responsible for both aberrant morphology and mitochondrial dysfunction observed in LRRK2^G2019S^ hiA. Abnormalities in neurites outgrowth and branching are among the earliest pathological phenotypes observed in LRRK2 mutations^61,62^. It has been initially proposed that the origin of these morphological changes could be related to an apoptotic process^62^; however, further studies provided evidence for an association of LRRK2 with tubulin/actin, thus suggesting that morphological changes may be consequences of LRRK2-modulation of cytoskeletal dynamics^63^. Several lines of evidence suggest the relationship of LRRK2 protein with the cytoskeleton: (i) The GTPase domain of LRRK2 protein can pull-down α/β tubulin from cell lysates of mouse fibroblasts and human embryonic kidney^64^; (ii) LRRK2 co-precipitates with β tubulin from wild-type mouse brain and (iii) Recombinant LRRK2 can phosphorylate β tubulin in vitro^65^. High-throughput screening of LRRK2 interactome revealed proteins of the actin family and of the actin-regulatory network as interactors of LRRK2 in actin polymerisation in vitro^16^. We presume therefore, that the atrophy observed in PD LRRK2^G2019S^ astrocytes could be a consequence of the mutated LRRK2 protein breakdown that becomes unable to properly modulate cytoskeletal dynamics. Similarly, LRRK2 mutation can be responsible for mitochondrial dysfunction and fragmentation, as already observed in fibroblasts, neural stem cells or neuroblastoma cell lines^13,66–68^. Multiple studies demonstrated that LRRK2 loss of function, associated with G2019S and R1441G mutations impair mitochondrial oxidative state increasing the neuronal susceptibility to oxidative stress damage^69–71^. One possible explanation might be a mitochondrial DNA damage induced by the LRRK2 mutations, which was observed in midbrain cultures and PD patient-derived lymphoblastoid cell lines^72^.

The observations that LRRK2 mutation may be responsible for morphological atrophy and mitochondrial malfunction, indicate possible mechanism associated with reduced neuroprotection in this mutation carriers. A recent observation demonstrated that G2019S mutation in hiA alters the astrocyte-to-neurone communication mediated by extracellular vesicles^73^. In this work, the LRRK2 mutation in astrocytes was claimed to affect morphology and the content of extracellular vesicles and multivesicular bodies (MVB). The authors found that neurones incorporated astrocyte MVB with an abnormal accumulation of key PD-related proteins such as LRRK2 and phospho-S129 α-Syn. Dopaminergic neurones incorporating the dysfunctional MVB released by the LRRK2^G2019S^ astrocytes showed an aberrant morphology^73^.

In this study, we propose an hallmark for PD with LRRK2^G2019S^ mutation. Our hypothesis postulates that astrocytes with this mutation fail to support neurones because of loss of homoeostatic support resulting from substantial morphological atrophy and loss of complexity; in addition, astrocytes demonstrated mitochondrial dysfunction that also affects their neuroprotective capabilities.

## Methods

### Human samples

Human fibroblasts were obtained from two healthy donors (Ctrl1 was purchased from AXOL and Ctrl 2 from the Coriell stem cell bank) and two Parkinson´s disease patients with LRRK2^G2019S^ mutation (PD1 from the Coriell stem cell bank and PD 2 provided by the BioDonostia Hospital, San Sebastian, Spain) (Supplementary Table 1). Control patients who matched PD donors in age and gender did not show any neurological symptoms. All procedures with human cells were approved by the National and local ethical committees (with code M30_2018_030) according to the National law 14/2007 on Biomedical research.

### Generation of human induced astrocytes (hiA)

*Fibroblasts* were grown in DMEM F12 (Gibco/ThermoFisher, Spain) and infected with the CytoTune iPS 2.0 Sendai Reprogramming Kit (Thermofisher, Spain) as described in Supplementary Fig. 1. The commercial Sendai virus expressed the key genetic factors necessary for reprogramming somatic cells into iPSCs (Klf4/Oct3-4/Sox2-KOS, hc-Myc, Klf4). Infection efficiency was evaluated by co-infection with a EmGFP fluorescent reporter plasmid provided by the kit. Seven days later, transduced fibroblasts were seeded in Geltrex (ThermoFisher, Spain) in Essential 8 Flex medium (E8, Gibco/ThermoFisher, Spain). E8 medium was changed every day for 21 days until we observed the formation of iPSC colonies. Colonies were manually isolated using a 27G Braun Sterican Needle and replated in laminin-521 (LN521-Biolamina, Sundbyberg Sweden) with E8 medium and ROCK inhibitor (Y-27632; Millipore, Madrid, Spain). The day after, ROCK inhibitor was removed and replaced with fresh medium. Colonies were sequentially isolated and re-suspended at single cell level. *Embryoid bodies* (EB) were generated (Supplementary Fig. 2) after re-suspending the iPSC colonies in Essential 6 medium (E6, Gibco) for 2–4 days in the AggrewellTM800 plates (StemCell, Grenoble, France). Half-medium in the microwells was replaced daily with fresh medium. EBs were then seeded in a LN521/LN211 mix (50% each) (Biolamina) and the differentiation to neural precursor cells (NPC) as neural rosettes was promoted using the STEMdiff Neural Induction Medium (Stemcell). After 7 days, neural rosettes were selected and detached using the STEMdiff Neural Rosette Selection Reagent (Stemcell). Cells were incubated for 2 h with this reagent at 37 °C with 5% CO_2_ and then, mechanically re-suspended at single cell level and seeded in LN211/LN111 (50% each) (Biolamina). *Differentiation* of NPC to progenitor astrocytes was triggered using the astrocyte differentiation medium (STEMdiff astrocyte differentiation #100-0013, StemCell). To maintain the appropriate cell density (70% of confluence) cells were passed every week in the same coating mix during 21 days. *Maturation*. Finally, astrocytes progenitor cells were maturated in the Astrocyte Maturation Medium (STEMdiff astrocyte maturation #100-0016, StemCell) for 60 to 75 days. During the whole protocol, the correct state of the cells in each step was evaluated using the EVOS FL microscope (Life Technologies, AME4300). See Supplementary Fig. 1 for an overview of simplified protocol steps.

### Cytofluorimetry assay

Cells (500.000) were detached with TryPLE (Sigma, Spain) and fixed as a single cell suspension with PFA 4% for 10 min. Cells were washed in phosphate-buffered saline (PBS) at 400 x *g* for 5 min and re-suspended in blocking solution (0.5 g BSA in PBS with 0.01% Triton (PBS-T) with agitation overnight at 4 °C. The following day cells were washed and incubated with the primary antibody goat anti-GFAP (Abcam, 53554) for 2 h at 4 °C. After further wash for 5 min in PBS-T 0.01% cell suspension was incubated with the secondary conjugated antibody Alexa fluor 488 donkey anti-goat for 1 h at 4 °C. After a further wash with PBS-T 0.01%, cells were finally re-suspended in PBS 1x. Cells were analysed in the BD FACSJazz (USB, inFlux Compact) analyser using the Blue 488 laser. Unstained cells were gated and used as a negative control.

### Calcium imaging

Cytosolic calcium (Ca^2+^) levels were estimated by the 340/380 ratiometric microfluorimetry as described previously^74^. Astrocytes were loaded with fura-2 AM (5 μM; ThermoFisher/Invitrogen) for 20 at 37 °C and subsequently washed in the recording solution containing 137 mM NaCl, 5.3 mM KCl, 0.4 mM KH_2_PO_4_, 0.35 mM Na_2_HPO_4_, 20 mM HEPES, 4 mM NaHCO_3_, 10 mM glucose, 1 mM MgCl_2_, 2 mM CaCl_2_ (pH 7.4) to allow de-esterification. In experiments with FCCP, Ca^2+^ was omitted from the recording solution. Experiments were performed in a coverslip chamber continuously perfused with buffer at 1 ml/min by exposing the cells to ATP (100 μM) or FCCP (1 μM). The perfusion chamber was mounted on the stage of a Zeiss (Oberkochen, Germany) inverted epifluorescence microscope (Axiovert 35), equipped with a 150 W xenon lamp Polychrome IV (T.I.L.L. Photonics, Martinsried, Germany), and a Plan Neofluar 403 oil immersion objective (Zeiss). Cells were visualised with a high-resolution digital black/white CCD camera (ORCA, Hamamatsu Photonics Iberica, Barcelona, Spain) and images were acquired every 5 s. Image acquisition and data analysis were performed using the AquaCosmos software programme (Hamamatsu Photonics Iberica). Intracellular Ca^2+^ measurements were expressed as the ratio of *F*_340_/*F*_380_ and normalised to baseline values. Results for statistical comparison were calculated as area under the curve (AUC) of the response for each cell from the start of each compound application.

### Immunofluorescence

Cell cultures were fixed in 4% para-formaldehyde (Merck/Sigma), permeabilised with 0.1% Triton (Sigma) and non-specific epitopes were blocked with 5% normal goat serum in PBS. Primary antibodies (Supplementary Table 2) were incubated overnight and then washed three times with 0.1% Triton in PBS. Secondary conjugated antibodies Alexa 488, Alexa 594, Alexa 647 or Alexa 405 (Invitrogen, 1:500), were incubated for 1 h in the dark at room temperature. After three washes with 0.1% Triton in PBS, cell nuclei were counter-stained for 1 min with DAPI (ThermoFisher). Finally, coverslips were mounted with Glycergel (Dako, Barcelona, Spain) and analysed using the confocal microscope Leica TCS STED CW SP8.

### Morphological analysis by *high-content screening*

Cells were seeded in glass bottom Cellvis 24-well plates (Cellvis, Bilbao, Spain) coated with LN111/LN211 (Biolamina). After fixation with 4% PFA for 8 min, cells were immunostained for GFAP expression (Goat anti-GFAP, Abcam 53554). Alexa fluor Donkey anti-goat was used as a secondary antibody. Images were taken with the CellInsight CX7 *high-content screening* system (Thermo Scientific) using a 10x objective. Morphological parameters for area (defined as the number of microns squared of the object), perimeter (length of the boundary of the object) and ShapeP2A (measure of the ratio of the perimeter squared of the object to four times) were calculated with High-Content Analysis platform. More than 100 cells per cell line were analysed.

### Morphological assessment

Sholl analysis was performed with the public software Fiji^75^, to measure the complexity of GFAP-positive human astrocytes. A transparent grid with concentric circles (every 5 μm from the centre of the cell soma across the whole radius) were superimposed onto the cells after immunofluorescence with GFAP antiserum. Sholl measurements were obtained by quantifying the number of intersections with each concentric circle.

### Electron microscopy

Cells were fixed in 4% PFA for 10 min and post-fixed in 3% glutaraldehyde for 30 min. After a wash in phosphate buffer (PB) samples were osmicated (1% OsO_4_ in 0.1 M PB; pH 7.4) for 30 min. After 3 x 10 min washes in 0.1 M PB, samples were dehydrated in graded ethanol concentrations (50% to100%) to propylene oxide and embedded in epoxy resin (Sigma-Aldrich) by immersion in decreasing concentration of propylene oxide (1:3 for 30 min, 1:1 for 1 h and 3:1 for 2 h). Samples were then embedded in fresh resin overnight and allowed to polymerise at 60 °C for 2 days. Following visualisation at the light microscope, selected portions were trimmed and glued onto epoxy resin capsules. Semi-thin sections (500 nm-thick were cut from epoxy blocks using a Power Tome ultramicrotome (RMC Boeckeler, Tucson, AZ, USA and stained with 1% toluidine blue. Ultrathin (50–60 nm thick) sections were then cut with diamond knife (Diatome, Hatfield PA, USA), collected on nickel mesh grids and stained with 4% uranyl acetate for 30 min and 2.5% lead citrate for electron microscope visualisation. For *Image Acquisition and analysis*, electron microscopy images of mitochondria were taken from randomly selected fields with a Jeol JEM 1400 Plus electron microscope at the Service of Analytical and High-Resolution Microscopy in Biomedicine of University of the Basque Country UPV/EHU. Images were taken at a magnification of 12,000X. Circularity and aspect ratio (ratio of circularity vs. elongation) were measured with Fiji-Software using a self-made plug-in. More than 100 mitochondria were analysed for each line.

### Mitochondrial membrane potential (ΔΨ_m_) measurement

Mitochondrial membrane potential (ΔΨ_m_) of human astrocytes was assessed by the Rhodamine 123 (Rh123) staining. Briefly, cells were seeded in 35 mm glass bottom plates (Ibidi GmbH, Germany) at a mean density confluence of 50–70% and loaded with 10 μM Rh123 at 37 °C and 5% CO_2_. After 15 min cells were washed with 900 μl Hanks’ balanced salt solution and analysed by time lapse every 15 s for 5 min using the confocal microscope Leica TCS STED CW SP8. To establish the basal line, cells were stimulated with 1 μM FCCP after the first 60 s. Fluorescence intensity after FCCP treatment was measured with the Leica LASX Software and data were analysed with GraphPad Prism 5 (San Diego, CA, USA).

### Measurement of mitochondrial function and glycolytic activity

The oxygen consumption rate (OCR), as an indicator of mitochondrial respiration, the extracellular acidification rate (ECAR), as indicator of glycolytic activity, and the proton efflux rate (PER), which correlates with lactate production, were measured with the Seahorse XF96 extracellular flux analyser. For the analysis of mitochondrial respiration, human astrocytes (30,000 cells/ mm^2^) were seeded in LN211/LN111 (Biolamina) precoated wells. The day of the experiment, cell medium was changed to Mito XF Medium (XF basal medium with phenol red, 0.001 M piruvic acid, 0.002 M glutamine, glucose 0.01 M, pH 7.4). The OCRs were obtained after the sequential treatment with oligomycin (2 μM), FCCP (1 μM), and rotenone combined with antimycin A (0.5 μM). To measure the *glycolytic activity*, we used the same protocol with the following modifications. The day of the experiment, cell medium was changed to Glico XF Medium without phenol red (DMEM Base Medium without Phenol Red with 5 mM HEPES, 10 mM glucose, 1 mM sodium pyruvate, 2 mM glutamine, pH 7.4 at 37 °C). The ECAR and PER were obtained after the sequential treatment with rotenone combined with antimycin A (0.5 μM) and 2DG (50 mM), respectively. Four replicates were performed for each condition or cell type for every experiments (*n* = 3). Data was analysed with the Wave 2.4.0 software.

### Western blot for detection of oxidatively modified proteins (oxyblot)

Astrocytes (30.000/well) were maturated for 60 days in 24-well plates coated with LN211/LN111 (Biolamina) and solubilised for 20 min with equal volume of 2x Extraction Buffer. Samples were prepared according to manufacturer’s instruction with all the reagents provided in Oxidised protein western blot detection kit (ab178020; Abcam). Briefly, the carbonyl groups in the protein side chains were derivatised to 2,4-dinitrophenylhydrazone (DNP-Hydrazone) by reaction with 2,4-dinitrophenylhydrazine (DNPH). Two aliquots of each sample were prepared to be analysed simultaneously. One aliquot was treated with “derivatisation reaction” (DNPH Solution) and the other control aliquot was treated with “derivatisation control reaction”. Protein concentration was quantified with a detergent-compatible assay reagent (Pierce BCA Protein Assay Kit) according to the manufacturer’s instructions (ThermoFisher Scientific). Proteins loading was normalised after Red Ponceau staining. All blots derive from the same experiment and they are processed in parallel.

### Statistical analysis

Results are expressed as mean ± standard error of the mean (S.E.M) with *n* corresponding to the number of cells or cultures tested. Data were analysed with Excel (Microsoft, Seattle, WA, USA) and GraphPad Prism software. Statistical significance between datasets was tested using one-way analysis of variance (ANOVA) followed by Newman–Keuls multiple comparison test, with a significance threshold of *p* < 0.05.

### Reporting summary

Further information on research design is available in the Nature Research Reporting Summary linked to this article.

## Supplementary information

Supplementary Information

Reporting Summary

## Data Availability

The data that support the findings of this study are available from the corresponding author upon reasonable request.
