# Supplementary material for: Astrocytic atrophy as a pathological feature of Parkinson’s disease with LRRK2 mutation
Source: NPJ Parkinsons Dis. 2021 Mar 30;7:31. doi: 10.1038/s41531-021-00175-w (PMC8009947; doi:10.1038/s41531-021-00175-w)
Supplement: Supplementary file 1 — Supplementary Information [file 41531_2021_175_MOESM1_ESM.pdf]

Sample	Type	Code	Biopsy	Sex	Age	Mutated gene	Mutation	Karyotype
**Ax0083**	Healthy	Ctrl1	Skin	F	Commercial	-	-	Normal
**38530A**	Healthy	Ctrl2	Skin	M	55	-	-	Normal
**PD33879**	PD	PD1	Skin	F	66	LRRK2	G2019S	Normal
**FH1509**	PD	PD2	Skin	M	58	LRRK2	G2019S	Normal

Supplementary Table 1. Description of healthy and PD donors. Ax0083 was commercially derived and didn’t show the age donor. All kariotype were normal, as evidenced in Fig S4

Primary Antibody	Reactivity	Reference	Dilution
**GFAP**	Rat, Human	Abcam (ab53554)	1:1000
**S100b**	Human	Dako	1:400
**EAAT2**	Human	Santa Cruz	1:1000
**CD49f**	Human	Biolegend	1:750
**SOX2**	Human	Santa Cruz (17320)	1:300
**Oct4**	Human	Santa Cruz (sc-5279)	1:100
**Nanog**	Human	Abcam (ab21624)	1:1000
**MAP2**	Human	Abcam	1:500
**NG2**	Human	Abcam (ab50009)	1:300
**βIII tubulin (Tuj I)**	Human	Thermo Fisher (A25538)	1:500
**Smooth muscle actin, SMA IgG2a**	Human	Thermo Fisher (A25538)	1:200
**a-fetoprotein, AFP IgG1**	Human	Thermo Fisher (A25538)	1:500

Supplementary Table 2. Primary antibodies used in the paper, with reference and working dilution

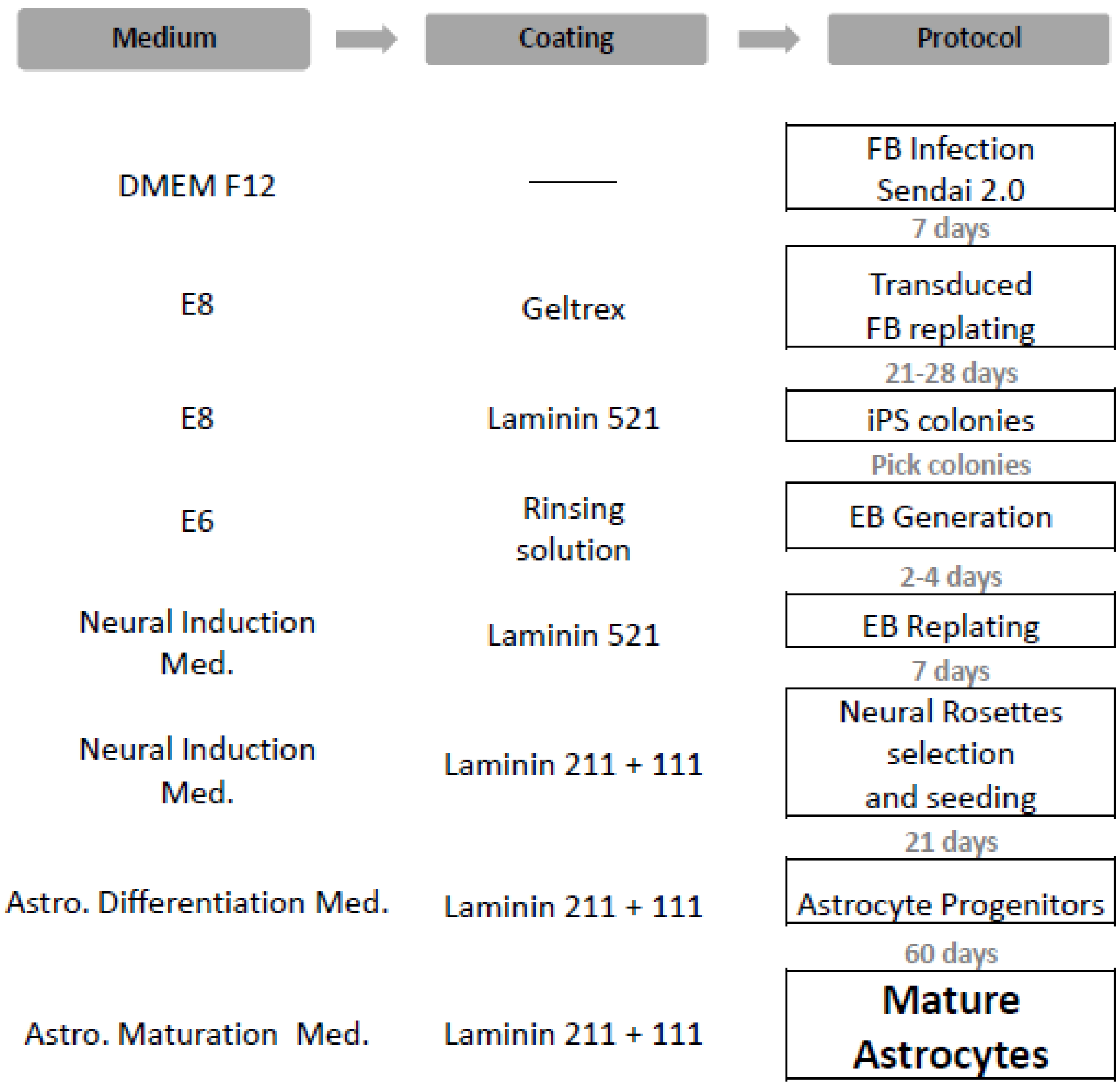

Supplementary Figure 1. Schematic view of the protocol used to reprogram and differentiate human fibroblasts to hiA.

Klf4–Oct3/4–Sox2 (KOS), L-Myc, and Klf4.

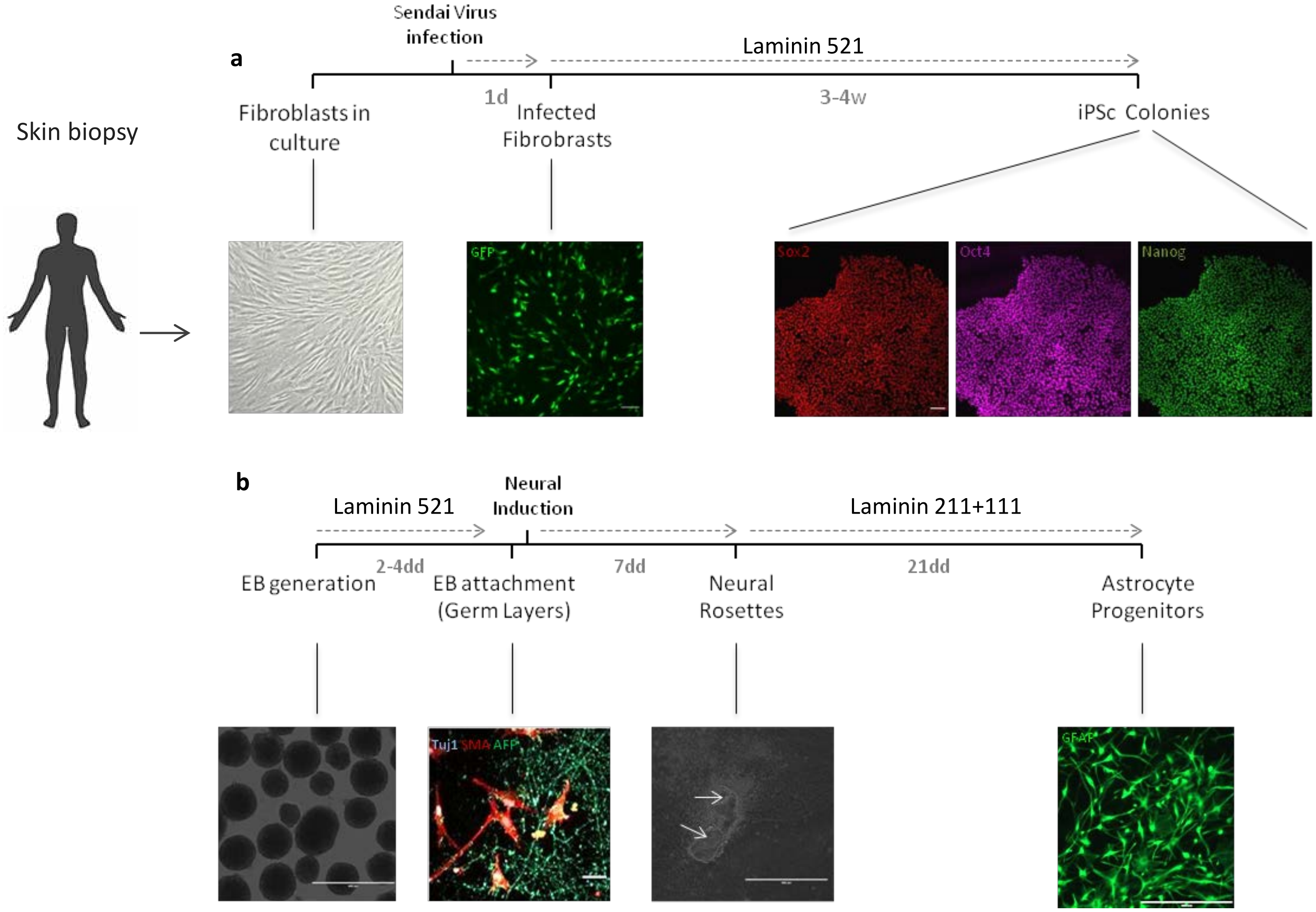

Supplementary Figure 2. Protocol and cell characterisation at different culture steps. Fibroblasts infection was evidenced by the co-infection with eGFP protein. iPSC colonies were positive for the pluripotent markers Sox2 (red), Oct4 (purple) and Nanog (green). Embryoid bodies (EB) spontaneously differentiated to the 3 germ layers as evidenced by Tuj1 (in blue for endoderm), SMA 8in red for mesoderm) and AFP (in green for ectoderm). Astrocyte progenitors were positive for GFAP immunostaining (green).

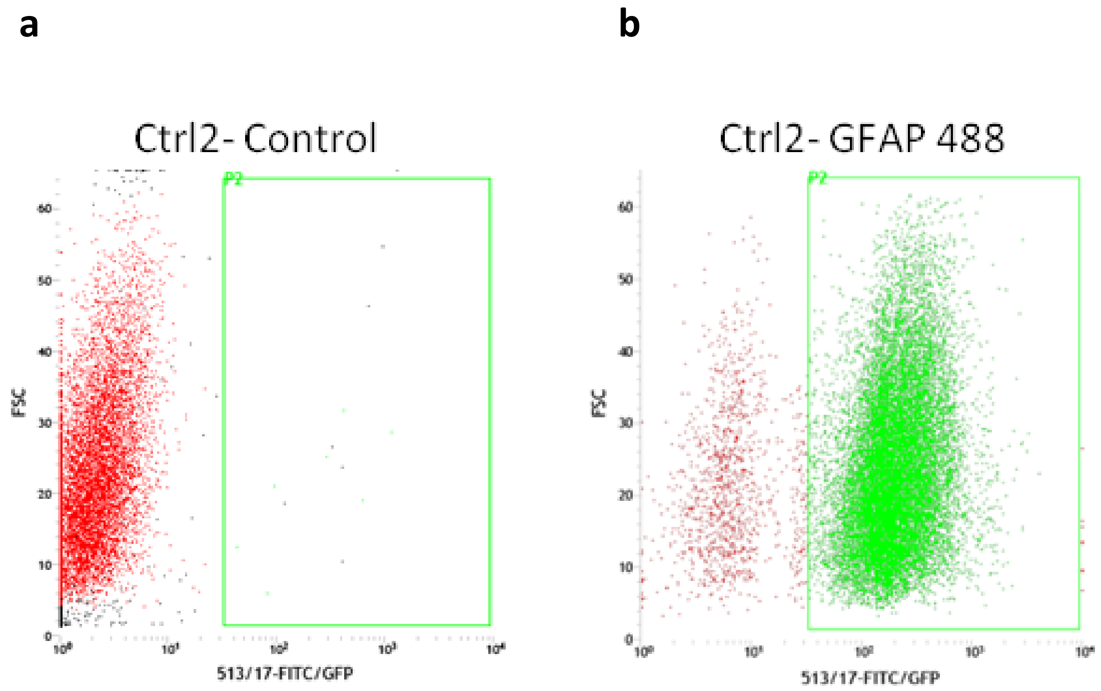

Supplementary Figure 3. **Astrocytes differentiation efficiency**. Representative cytofluorimetry assay of healthy donors. hiA were maturated for 60 days in vitro, fixed with 4% PFA (as described in Material and Methods) and stained with anti-GFAP. Unstained cells are visualised in A whereas specific stained cells are gated in B.

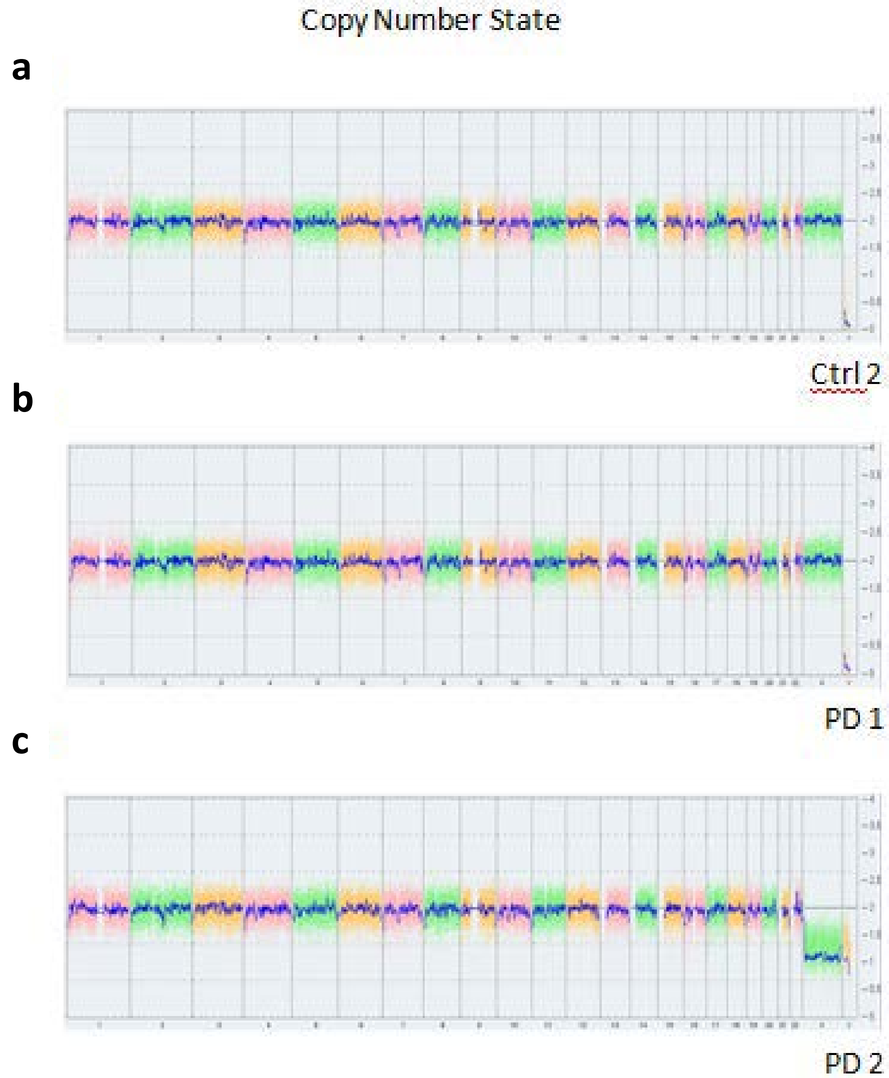

Supplementary Figure 4. Whole genome view. The whole genome view displays all somatic and sex chromosomes in one frame with high level copy number. The smooth signal plot (right y-axis) is the smoothing of the log2 ratios that depict the signal intensities of probes on the microarray. A value of 2 represents a normal copy number state (CN = 2). A value of 3 represents chromosomal gain (CN = 3). A value of 1 represents a chromosomal loss (CN = 1). The pink, green and yellow colors indicate the raw signal for each individual chromosome probe, while the blue signal represents the normalised probe signal which is used to identify copy number and aberrations (if any). A, B and C correspond to Ctrl 2, PD 1 and PD 2 respectively, and do not show any alteration. Ctrl 1 were not analyzed because of commercial derivation and already tested by Axol.

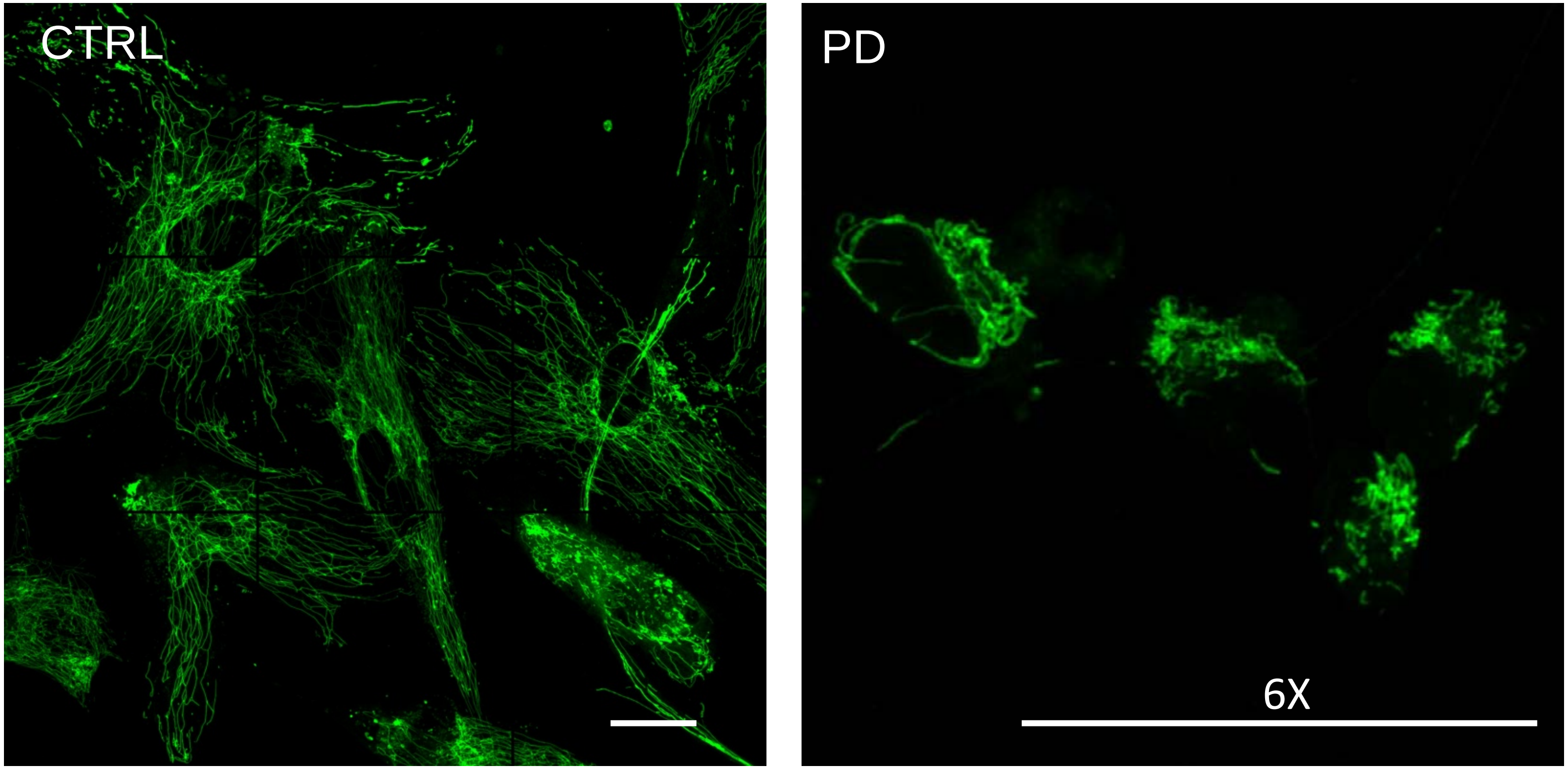

Supplementary Figure 5. Mitotracker staining. Healthy (Ctrl) and patient (PD)-derived astrocytes were seeded in laminin 111/211 coated 35mm glass transparent wells. After 30 day of maturation, astrocytes were loaded in vivo with 100nM Mitotracker green probe for 30 minutes, washed and analysed by confocal microscopy. Scale bar represents 20μm.

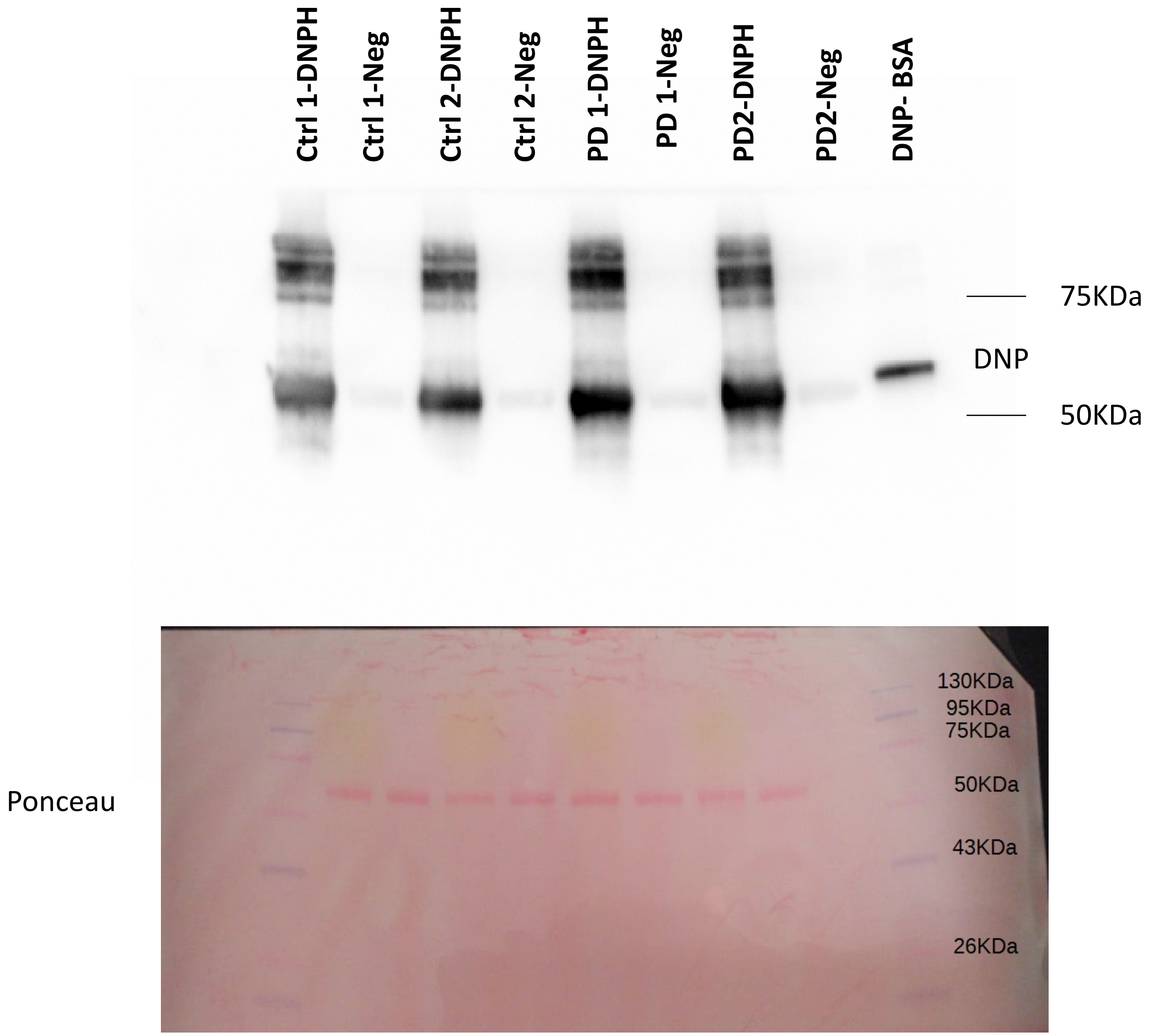

Supplementary Figure 6. Original Oxyblot. Original, uncropped Oxyblot and ponceau used to normalize DNPs
